# Impact of malnutrition management e-learning module on GPs’ knowledge: a pilot study

**DOI:** 10.3399/BJGPO.2022.0111

**Published:** 2023-01-11

**Authors:** Aisling A Geraghty, Patricia Dominguez Castro, Ciara ME Reynolds, Sarah Browne, Frank Bourke, Catriona Bradley, Karen Finnigan, Sarah Clarke, Barbara Clyne, Gerard Bury, Carla Perrotta, Sharon Kennelly, Clare A Corish

**Affiliations:** 1 School of Public Health, Physiotherapy and Sports Science, University College Dublin, Dublin, Republic of Ireland; 2 UCD Institute of Food and Health, University College Dublin, Dublin, Republic of Ireland; 3 Irish Institute of Pharmacy, Royal College of Surgeons in Ireland, Dublin, Republic of Ireland; 4 Health Service Executive Medicines Management Programme, Trinity Centre for Health Sciences, St James’s Hospital, Dublin, Republic of Ireland; 5 Department of General Practice, RCSI University of Medicine and Health Sciences, Dublin, Republic of Ireland; 6 School of Medicine, University College Dublin, Dublin, Republic of Ireland; 7 National Primary Care Division, Community Funded Schemes Service Improvement, County Laois, Republic of Ireland

**Keywords:** malnutrition, education, medical, continuing, oral nutritional supplements, general practice, primary healthcare

## Abstract

**Background:**

Malnutrition is underdiagnosed in primary care. GPs are key healthcare contacts for older adults at risk of protein-energy malnutrition; however, lack of knowledge and confidence in its diagnosis and treatment is often reported.

**Aim:**

To evaluate the impact of a bespoke online education module on GP malnutrition knowledge and management.

**Design & setting:**

A prospective pre—post pilot study with 23 GPs and eight GP trainees in the Republic of Ireland.

**Method:**

The module included units on the following: ‘malnutrition definition, prevalence, and latest evidence’; ‘identifying malnutrition in clinical practice’; ‘food-first advice’; ‘reviewing malnutrition’; and ‘oral nutritional supplements’. Participant knowledge was measured using a multiple choice questionnaire (MCQ) before and after the module (*n* = 31), and 6 weeks following completion (*n* = 11). Case studies assessing identification and management of malnutrition were evaluated by a clinical specialist dietitian with expertise in managing malnutrition. Changes in assessment performance were calculated using paired *t*-tests. Acceptability was evaluated using a questionnaire.

**Results:**

Post-training, 97% of GPs increased MCQ scores from baseline (+25%, *P*<0.001), with the greatest improvement in ‘identifying malnutrition in clinical practice’ (mean increase 47%, *P*<0.001). Eleven GPs completed the 6-week MCQ with scores remaining significantly higher than baseline (mean increase 15%, *P* = 0.005); ‘identifying malnutrition in clinical practice’ remained the most highly scored (mean increase 40%, *P*<0.001). Seventeen GPs completed the case studies; 76% at baseline and 88% post-module correctly calculated malnutrition risk scores. Appropriate malnutrition management improved for 47% of GPs after module completion.

**Conclusion:**

This e-learning module improved malnutrition knowledge, with good short-term retention in a small cohort. Development of online evidence-based nutrition education may improve GP nutrition care.

## How this fits in

GPs receive limited under- or post-graduate education on nutrition. An online e-module on the identification and management of malnutrition was designed by dietitians, with the content guided by GP interviews and community-based healthcare professional (HCP) focus groups. GPs who completed the e-module significantly improved their theoretical knowledge and skills in identifying and managing malnutrition.

## Introduction

Risk of protein-energy malnutrition increases in older adults due to the physiological, functional, and psychosocial changes that occur with ageing.^1^ Global data indicate that between 5% and 10% of older adults living at home, and 20% in residential care settings, are at risk of protein-energy malnutrition.^2,3^ Older adults typically consult their GP with concerns about weight loss or poor appetite;^4,5^ however, GPs may not routinely screen for or prioritise the management of malnutrition.^6^

Key aspects of best-practice nutrition care to diagnose malnutrition are to screen patients for risk using a validated tool, then ensure treatment, if required, is initiated and monitored.^7^ Lack of nutrition training and time constraints have been widely reported as major barriers to the identification of malnutrition among HCPs.^5,8–10^ In previous research, HCPs have reported that they struggled to identify malnutrition, did not feel sufficiently skilled to carry out nutritional assessment, or organise nutrition care.^11,12^ Similar findings were reported among Irish GPs, who reported a lack of knowledge and confidence to identify and treat malnutrition.^6^

HCPs acknowledge the importance of nutrition and the inadequacy of nutritional training.^11,13^ A previous dietetic-led education programme for HCPs in primary care reported improved malnutrition screening, first-line dietary advice, and referral to dietetic services 1 year after the intervention.^14^ Preference towards the availability of online rather than face-to-face education and/or training has been expressed by Irish GPs.^15^ There is a need, therefore, to improve accessibility to evidence-based nutrition education material online.^16^

Using existing literature,^17^ and informed by data from interviews and focus groups with GPs, HCPs, and patients with malnutrition,^5,6,18,19^ an online e-learning module was developed. This module focused on the identification and management of malnutrition in the community and primary care setting aimed at GPs and GP trainees. It applied principles of instructional design to enhance online learning experience. The aim was to evaluate the effectiveness of the module in improving GP knowledge, reported practice, and decision-making around malnutrition identification and management.

## Method

### Setting and participants

This was a prospective pre—post pilot study, with a convenience sample of 23 GPs and eight GP trainees in the Republic of Ireland, which was conducted from August–October 2020. GPs were recruited via social media, and professional and university networks. Participants did not receive financial reimbursement for participating in the project; however, the module was eligible for continuing professional development (CPD) credits and participants were entered into a draw to win a €150 gift voucher. More information on recruitment of GPs and HCPs into this project (ONSPres Study) has been published.^20^

### e-Learning module development and content

The module content was developed by a multidisciplinary collaborative, which was led by research dietitians, and informed by qualitative interviews and focus groups with GPs and HCPs.^5,6,15^ GPs expressed a preference for online malnutrition education owing to their busy work schedules.^6,15^ The GPs and community pharmacists also suggested the following: CPD points on completion of the programme would incentivise engagement; the programme should contain downloadable and printable low-literacy patient information; the content should be clearly displayed using flowcharts or algorithms; and it should include an online quiz and case studies.^5,6^ Specific content preference themes requested by GPs included the following: guidance on available oral nutritional supplements (ONS) and the differences between them; how to discontinue, change, and review ONS; dietary and food-first advice; ONS for specific diseases; and appropriate ONS use. All healthcare professions expressed the need for a care pathway for malnutrition, including a referral process to community dietitians and patient information leaflets.^5^ All groups also reported needing information on the types of ONS available and the differences between products.

Instructional designers adapted Gagné’s model of instructional design and his 'events of instruction' to motivate and engage the audience. They did this by gaining attention via the programme’s style and imagery, stimulating recall or prior learning by building on the GPs’ existing knowledge, providing learner guidance, and by assessing performance via knowledge checks and quizzes. The content design included authentic learning 'tasks' to enable course participants to make connections to real-life issues and their experiences.^21^ They also used realistic ONS-related 'activities',^22^ which are designed to help GPs practise skills in situations similar to those where the skills will be used via scenarios, cases, or problem-based interactions. ONS-related tasks and activities were based on the outputs from the semi-structured interviews and focus groups,^15^ and in collaboration with the programme’s subject-matter experts and training content developers. The e-module content, assessments, and satisfaction survey were reviewed by the GPs on the research group (GB and CP), by two of the original GP interviewees, and by the GP representative at the Irish Health Service Executive (DH) who was also a member of the research group.

**Figure 1.**
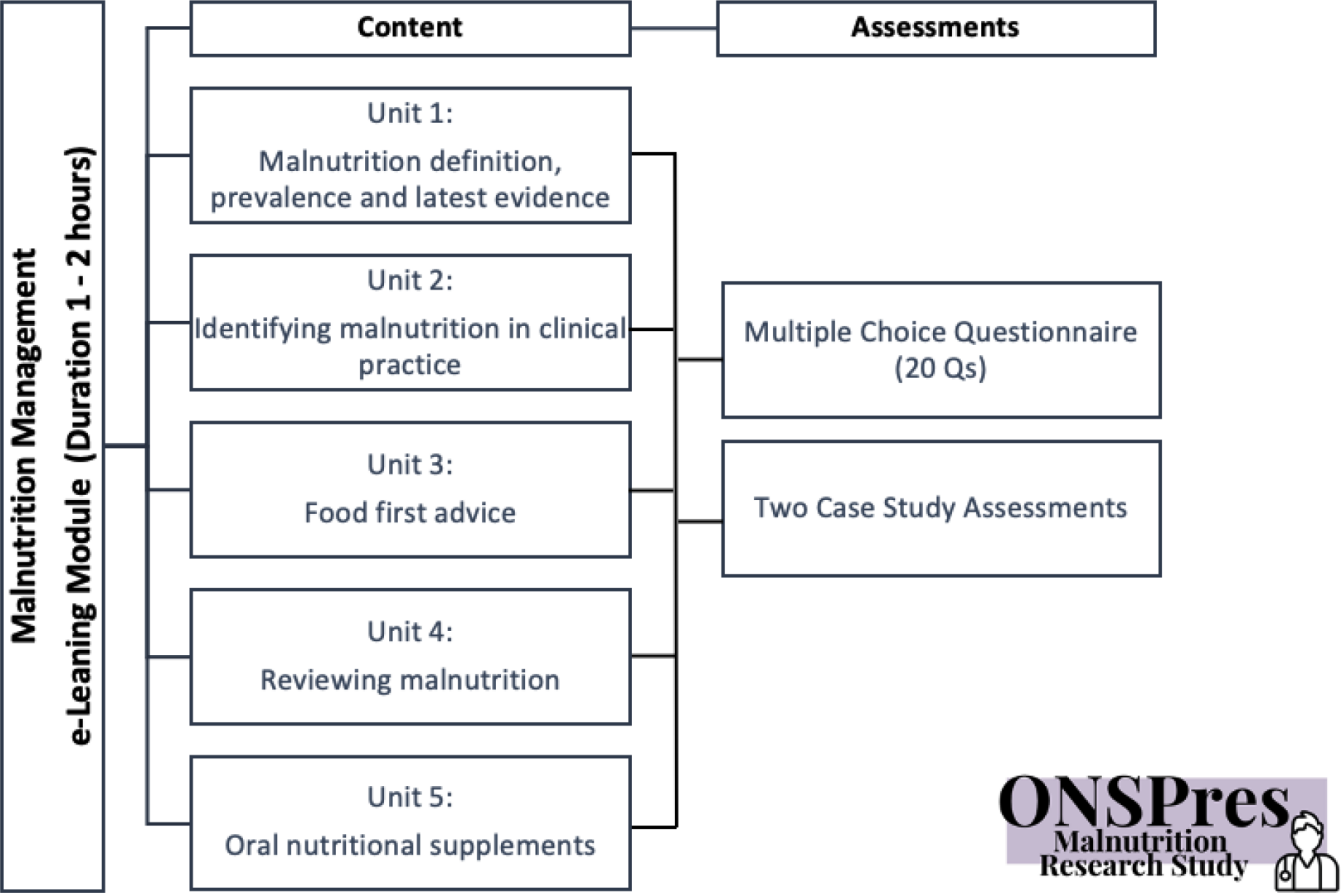
Malnutrition management e-learning module overview

The module covered units on the following topics: ‘protein-energy malnutrition definition, prevalence and latest evidence’; ‘identifying malnutrition in clinical practice’; ‘food-first advice’; ‘reviewing malnutrition’; and ‘oral nutritional supplements’, and took approximately 2 hours to work through. GPs had unlimited access to the module to enable them to complete it in their own time. Module structure is described in Figure 1.

The assessment included a 20-item, evenly weighted, multiple-choice questionnaire (MCQ) (Supplementary Table S1) and two patient case studies (Supplementary Figure S1) developed by the research team. After completing the training, GPs were invited to immediately repeat the assessments, and again 6 weeks later.

### Outcomes

Baseline, post-completion, and follow-up MCQ scores were compared to assess changes in knowledge. The MCQ is provided in Supplementary Table S1. The two patient case studies consisted of nine questions each, which explored calculations of body mass index (BMI; kg/m²), weight loss percentage, and malnutrition screening tool scores (using the Malnutrition Universal Screening Tool [MUST]). Participants had to decide if the patient was malnourished or at risk of malnutrition, list appropriate malnutrition screening tools, and explain their approach to treatment and follow-up. Participant answers were compared with an ‘optimum’ answer for each case study that was developed by a registered clinical specialist dietitian with expertise in the management of malnutrition. GP explanations for their approach to treatment and follow-up were also evaluated. The most frequent aspects of patient management within the GP answers were also summarised. Acceptability of the module was assessed through evaluation questionnaires with participants, using both closed and open questions.

### Statistical analysis

Statistical analysis was carried out using SPSS (Statistical Package for the Social Sciences) software (version 26.0). Descriptive statistics, including mean, median, and frequencies were generated as appropriate. Differences in knowledge between qualified GPs and GP trainees were investigated to explore whether experience level may influence knowledge. This was evaluated pre-completion, post-completion, and at follow-up using independent samples *t*-tests. Changes in overall and module-specific knowledge were assessed using paired samples *t*-tests. The level of significance was set at *P*<0.05.

## Results

Thirty-one (23 qualified and eight GP trainees) GPs completed the module and the MCQ assessment, with 17 also completing the case studies. A sub-sample of 11 GPs (eight GPs, three GP trainees) completed the 6-week follow-up MCQ, on average 8.8 weeks after module completion (standard deviation [SD]: 17.9, range 5.1–12.3 weeks). Mean baseline MCQ score was 63% (SD 10.62%). GPs had a mean score of 65% (SD 8.98%) and trainees had a mean score of 57% (SD 13.08%), *P* = 0.054.

**Figure 2.**
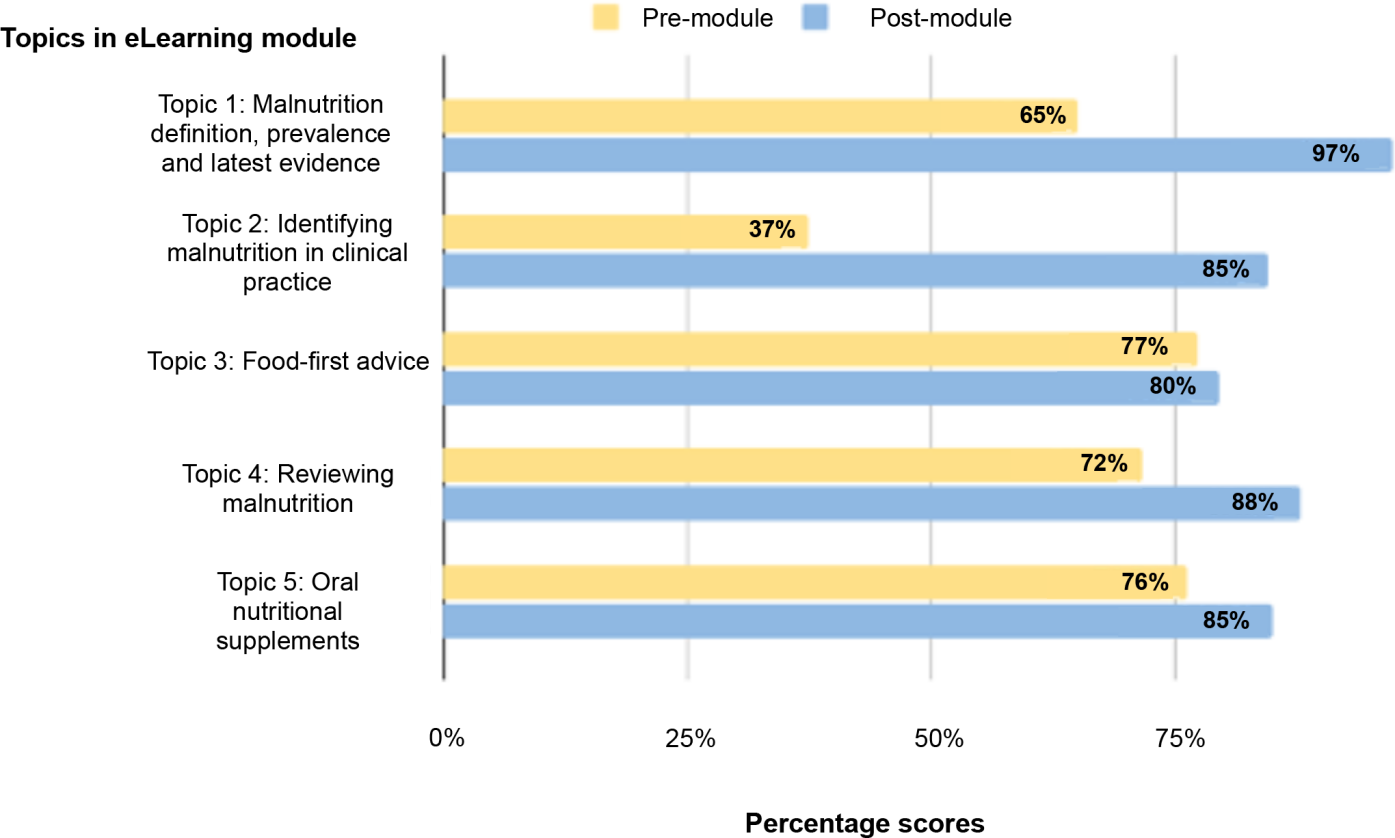
Mean GP multiple-choice questionnaire score (*n* = 31) across module units at baseline (pre-module) and immediately post-completion of malnutrition e-learning module

Thirty GPs (97% of the group) had higher MCQ scores after completing the module. MCQ scores significantly increased post-module with a mean score of 88% (SD 11.82%), and a mean increase of 25% (95% confidence interval [CI] = 20.14 to 29.22; *P*<0.001). GP trainees had a greater improvement in MCQ score compared with qualified GPs (34% improvement, SD: 10.61% versus 22% SD: 11.52%; *P* = 0.013). Mean scores for each MCQ question at baseline and post-module are detailed in Supplementary Table S1. At baseline, just one GP was able to correctly identify the most appropriate tool to screen for malnutrition; all 31 GPs were able to do so after completing the module (Supplementary Table S1). Before module completion, six GPs knew the most common manifestation of malnutrition. This increased to 26 GPs post-completion (Supplementary Table S1). The biggest improvement in MCQ score across the units was in ‘identifying malnutrition in clinical practice’. At baseline, the mean score in this unit was 37%, which increased to 85% post-module. Figure 2 outlines changes in MCQ unit scores from before to having completed the e-learning module.

The mean GP 6-week follow-up MCQ score was 82% (SD: 14.13), which remained significantly increased from baseline (mean increase 15%, SD 13.23; 95%CI = 5.26 to 22,01; *P* = 0.005). However, overall score in the follow-up MCQ had reduced from the scores achieved immediately post-module (mean decrease 10%, SD 13.96; 95%CI = 4.21 to 19.38; *P* = 0.039). Scores decreased for ‘malnutrition definition, prevalence, and latest evidence’ module (–11%) and ‘identifying malnutrition in clinical practice’ module (–13%) but did not revert to baseline. Scores for ‘reviewing malnutrition’ (–11%) and ‘oral nutritional supplements’ (–21%) at the 6-week follow-up were lower than baseline scores for the 11 participants. The score for ‘food-first advice’ module further increased (mean +9%) from post-module to the 6-week follow-up.

### Case studies

Seventeen GPs completed two patient case studies, which were presented before and after completion of the e-learning module. Case study 1 presented a patient at risk of malnutrition while case study 2 was a more severe case of malnutrition (Supplementary Figure S1). BMI and weight loss were calculated correctly at baseline and post-completion of the training. Correct calculation of MUST score for case study 1 increased from 94% (*n* = 16/17) of GPs at baseline to 100% (*n* = 17/17) post-module, and for case study 2 from 76% (*n* = 13/17) to 88% (*n* = 15/17). Only 59% (*n* = 10/17) of GPs were able to identify another appropriate screening tool at baseline; this increased to 94% (*n* = 16/17) post-module, with the ‘DETERMINE your nutritional health’ being the most frequently suggested alternative on both occasions.

### Clinical specialist dietitian review of case studies

The proportion of GPs who included all key components required to manage malnutrition in case study 1 increased from 71% (*n* = 12/17) at baseline to 82% (*n* = 14/17) post-completion and for case study 2, from 6% (*n* = 1/17) at baseline to 53% (*n* = 9/17) post-completion. GPs had good awareness of appropriate follow-up times with the correct timeframe being identified by all GPs for the patient in case study 1. For case study 2, 88% (*n* = 15/17) of GPs gave the correct timeframe pre-completion, which increased to 100% of GPs post-module completion.

For case study 1, provision of dietary advice was the most frequently reported approach to treatment both at baseline and post-module completion. Most GPs would recheck weight or BMI at the follow-up appointment, and 65% (*n* = 11/17) stated that they would question the patient about their diet. For patients no longer at risk of malnutrition, at baseline, 41% (*n* = 7/17) of GPs stated that they would reinforce dietary intake in their treatment plans. This increased to 76% (*n* = 13/17) of GPs reporting this approach post-module.

For case study 2, more GPs included dietary advice, ONS prescription, and referral to a dietitian in their patient management approach following completion of the e-learning module. Most identified the need to check weight and/or BMI, review diet, and rescreen at a follow-up appointment. If risk of malnutrition was no longer evident at the follow-up appointment, 65% (*n* = 11/17) of GPs stated they would reinforce dietary advice having completed the module, compared with only 29% (*n* = 5/17) stating this at the baseline assessment. While previously no GPs reported that they would review malnutrition risk at every subsequent appointment, 35% (*n* = 6/17) stated they would rescreen for risk at subsequent appointments after completing the e-learning module.

### Acceptability

Completing the e-learning module required approximately 2 hours. Twenty-five participants completed an evaluation questionnaire. 80% (*n* = 20/25) found the time required ‘about right’, 12% (*n* = 3/25) found it too long (with one reportedly spending 3.5 hours), and 8% (*n* = 2/25) were unsure. Comments on time investment varied with one participant recommending more time dedicated to *'prescribing different types of ONS, e.g, how to select which protein content'* and another recommending information to be *'relayed in less than 1hour total'*. The most useful aspect was how to diagnose malnutrition, followed by food-first advice, signposts to available online resources, and information about ONS. The least useful aspects were calculating BMI and repeating MCQs. The online format worked and was considered *'user-friendly*' and *'excellent in parts'*, with mixed views from a minority on the interactive nature, whereby some explicitly liked or disliked clicking through extra links or boxes to follow learning.

## Discussion

### Summary

In this relatively small pilot study, 23 GPs and eight GP trainees undertook a CME e-learning module online on the topic of identification and management of protein-energy malnutrition. The analysis of baseline and post-module completion assessments indicated an improvement in GP knowledge and theoretical management of malnutrition in primary care in this cohort. While baseline scores in the treatment section of the MCQ were not very low, potentially owing to GPs’ experiences with patients coming from hospital settings, key improvements were made around identifying malnutrition. In the smaller group (*n* = 11) who completed the 6-week follow-up assessments, improvement in knowledge was retained for most elements, although some reductions from immediately post-module were noted. The e-learning module design was acceptable to GP participants, who reported finding the module useful, particularly highlighting important aspects of the content being the information on screening tools and risk identification. They found the case studies realistic and helpful, and liked the additional resources that were linked in the units. Eighty per cent reported it being a suitable length.

Strengths and limitations

A major strength of this study is that the e-learning module content was based on a needs analysis conducted through interviews with GPs and HCPs, and that registered and clinical specialist dietitians were involved in the module design. The sample of 31 was considered adequate for this pilot study; however, the high attrition rate from post-module completion to follow-up assessment meant that the original cohort may not have been well represented, and follow-up findings need to be interpreted with caution. It also points to a challenge in evaluating long-term knowledge retention and integration of learning into practice. As this was a convenience sample of GPs who were able and willing to give their time to this research, selection bias cannot be ruled out and these GPs may not be representative of wider populations. Unfortunately, owing to time restraints, demographic details were not collected from the GPs so representativeness of the cohort could not be investigated. Selection bias should also be considered with regard the reduced number of 25 GPs completing the acceptability questionnaire and 11 GPs completing the 6-week follow-up MCQ. Follow-up times for the 6-week MCQ varied from 5–12 weeks post-module, so this variability should also be considered alongside the interpretation of the results. It must be acknowledged that GPs were recruited and completed the study during the COVID-19 pandemic, at its height in Autumn 2020, and that this is likely to have influenced participation rates in the study.

### Comparison with existing literature

CME is essential for GPs to keep abreast of developments in evidence-based medicine and health care. CME delivered online offers many advantages to HCPs in primary care, including timely access, temporal and geographic flexibility, reduced cost, convenience, and options to revisit learning resources.^23–25^ Demand continues to increase, particularly during the COVID-19 pandemic. Evidence suggests that learning outcomes for online CME are equivalent to live CME, delivering the same quality content.^23,26^ As online platforms develop, there are more opportunities for the use of interactive learning — for example, realistic clinical, patient-focused case studies — that appeal to a range of learning styles. One of the strengths of this malnutrition e-learning module reported by GPs was the inclusion of case studies that represent different levels of malnutrition risk that they often encounter in primary care. This also allowed for the evaluation of improved application of knowledge in patient management. CME that combines knowledge updates and interactive tasks is recognised as having the greatest impact on clinical performance.^27^

Many global studies highlight inadequate nutrition training among medical students and graduates.^28,29^ Given curricular time restraints within already packed medical education programmes, this study demonstrates that nutrition education needs can be met with online modules focused on clinical practice needs. Online training has been shown to be as effective or superior to in-person training for physicians.^26^ The need for GPs to identify and manage malnutrition is acknowledged owing to the likelihood of them encountering high-risk cases in practice.^2,3^ In addition, GPs in the Republic of Ireland, in common with GPs in many countries, are the primary prescribers of ONS, designed for the treatment of malnutrition when a food-first approach alone has not worked.^7,30^ The demand for further education, either face-to-face or online, does exist and it is vital that it is well-designed and evaluated.^25,29^

### Implications for research and practice

Improved knowledge about malnutrition screening tools will aid identification and management of malnutrition, which has important positive implications for public health and the nutritional care provided by GPs in primary care. Health-related quality of life among older adults can be improved and health and economic costs associated with malnutrition in primary care reduced.^31^ In addition, patient awareness of malnutrition risk from their GP may result in more timely care and improved shared decision-making.^32^ The availability of the e-module is particularly timely with the current shift to remote modalities of CME. In addition to improving access, incorporation of CME into the workday or protected time needs to be considered to fully allow HCPs to keep their knowledge updated. While this e-module focused on the identification and management of protein-energy malnutrition only, other related topics, such as malnutrition in obesity or paediatric malnutrition, also require further education and could perhaps be delivered in a similarly designed online module.

Future research should evaluate the e-module for learning and application to practice using a nationally representative, longitudinal study design. Information from the MCQ scores and case studies, and feedback within the acceptability questionnaire were used to revise this e-module. A case study was included with an example of appropriate screening and treatment to clarify methods and some additional resources were added to aid long-term knowledge retention of information. The group plans to make the e-module more widely available and to evaluate interest and impact in a larger cohort.

In conclusion, the malnutrition education e-module evaluated in this study is a realistic, clinically focused, online CME that GPs can undertake, which provides an opportunity for evidence-based professional development. The e-module addresses established GP learning needs, which resulted in improved knowledge on the identification and management of malnutrition in primary care in this small cohort. Further research is needed to confirm these results in a powered, representative cohort, and to evaluate impact on clinical practice.

## Data

The dataset relied on in this article is available from the corresponding author on reasonable request.
